# Study of the Influence of Gas Tungsten Arc (GTA) Welding on the Microstructure and Properties of Mg–Al–RE-Type Magnesium Alloys

**DOI:** 10.3390/ma18143277

**Published:** 2025-07-11

**Authors:** Katarzyna N. Braszczyńska-Malik

**Affiliations:** Department of Materials Engineering, Faculty of Production Engineering and Materials Technology, Czestochowa University of Technology, 19 Armii Krajowej Ave., 42-200 Czestochowa, Poland; k.braszczynska-malik@pcz.pl or kacha@wip.pcz.pl

**Keywords:** magnesium alloy, aluminium, rare-earth elements, gas tungsten arc (GTA) welding, microstructure and microhardness

## Abstract

The effects of the gas tungsten arc (GTA) welding process on the microstructure and microhardness of two Mg-5Al-3RE and Mg-5Al-5RE experimental alloys (RE—rare earth elements) are presented. Both alloys were gravity-cast in a steel mould and GTA-welded in the same conditions. Analyses of the alloys’ microstructure were carried out by scanning electron microscopy (SEM+EDX) as well as X-ray diffraction (XRD). In as-cast conditions; both alloys were mainly composed of α-Mg; Al_11_RE_3_; and Al_10_RE_2_Mn_7_ intermetallic phases. Additionally; α+γ eutectic (where γ is Al_12_Mg_17_) in the Mg-5Al-3RE alloy and an Al_2_RE phase in the Mg-5Al-5RE material were revealed. The same phase composition was revealed for both alloys after the GTA welding process. The results of the dendrite arm size (DAS) and Vickers microhardness measurements were also described. Both welded materials exhibited an intensive size reduction of the structural constituents after GTA welding. About 75% smaller values of the dendrite arm spacing were revealed in the fusion zones of the investigated materials than in the as-cast conditions. The GTA welding process also influenced the microhardness of the experimental alloys and increased them by about 21% compared to the base metal; which was the consequence of the refinement of the structural constituents.

## 1. Introduction

**Figure 1:**
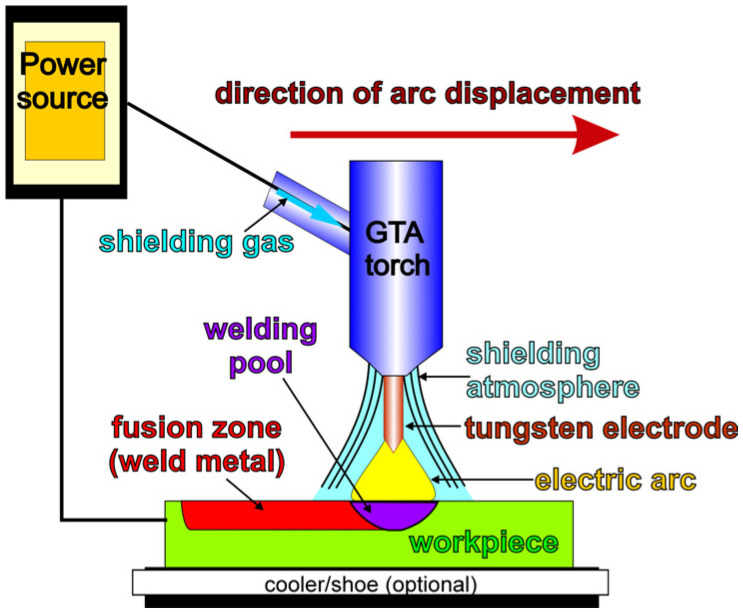
Scheme of gas tungsten arc (GTA) welding.

The development of magnesium alloys forces the search for modern technical solutions that cause a change in their microstructure and properties. Among them, welding technologies including laser welding [1,2,3,4,5,6,7], friction stir welding [8,9,10,11,12], tungsten inert gas (TIG) welding [13,14,15], or electron beam welding [16,17] processes have been recently widely investigated for magnesium alloys. Gas tungsten arc (GTA) welding (presented schematically in Figure 1) deserves special attention due to its economy and utility. This process is characterised by a flexibility of operation and short processing time, which results in time and energy saving, as well as processing precision [18,19,20,21]. The relatively small cost of equipment necessary for the GTA welding technique is also an undeniable advantage of using this process. Additionally, thanks to the very good quality of welds obtained in products after GTA welding, complications arising from weld defects such as weld spatter, porosity, and undercuts are minimized [22,23,24,25]. On the other hand, the fusion zone geometry can be designed thanks to the wide range of possible changes to the GTA welding parameters, which directly affects linear energy. Additionally, the choice of suitable welding parameters allowed the achievement of different size reductions of the microstructure in the fusion zone, which depends on the solidification conditions, especially the cooling rate [26]. It should also be added that magnesium alloys especially require special care during welding processes because of their high reactivity. Although shielding gas is typically used during the GTA process, its effect on the fusion zone in magnesium alloys has also been reported. Argon, helium, hydrogen, and nitrogen are used very often for GTA welding. Nevertheless, Marya et al. [27] concluded that helium, as a consequence of its high first ionization potential (24.6 eV), increases the constant-current voltage and creates a larger fusion zone than argon (15.6 eV).

Although there are several reports [1,18,28,29,30,31] describing the influence of GTA welding technology on magnesium alloys, the large majority of those investigations were concerned with commercial magnesium. In the presented work, the effect of the GTA welding process on the microstructure of two experimental Mg–Al–RE-type (RE—rare earth elements) alloys is presented. Even though aluminium constitutes a very important alloying element owing to its low price and advantageous effect on the strength properties or corrosion behaviour of magnesium alloys, it does not provide high-temperature strength, especially the poor creep properties of these alloys [32]. This set of properties results from the presence of a γ phase in the microstructure of Mg–Al-type alloys. The γ phase (also sometimes called β phase) is an intermetallic compound with an α-Mn-type cubic unit cell and a stoichiometric composition of Mg17Al12 (at 43.95 wt% Al). In the microstructure of Mg–Al-type alloys, a fully or partially divorced α+γ eutectic (where α is a magnesium solid solution) forms at a relatively low temperature equal to 710 K [32]. In order to especially improve the high-temperature properties of magnesium alloys, several alloy systems, focused on Mg–Al–third metal alloys, have been developed [33,34,35,36,37,38]. In Mg–Al–RE-type alloys, the γ phase is suppressed by the formation of mainly Al11RE3 (especially advantageous) and Al2RE intermetallic compounds. The real phase composition of an Mg–Al–RE-type alloy depends on the Al/RE ratio in its chemical composition and solidification conditions. These alloys are characterised by higher mechanical properties (especially after the high-pressure die casting process), including higher creep properties than those of Mg–Al-type alloys. They have also been investigated as a matrix alloy for magnesium composites [38,39,40]. It should also be noted that there are several reports describing the microstructure and properties of different Mg-Al-RE-type alloys based on single rare earth elements (Ce, La, Nd, Pr, etc.) [41,42,43,44,45,46]; however, using rare earth elements in the form of mischmetal is more economical. Additionally, it was reported [2] that rare earth elements reduced the tendency for porosity and cracking in magnesium castings. The main aim of this study is to analyse the microstructure and microhardness of two experimental cast Mg-5Al-3RE and Mg-5Al-5RE alloys after the GTA welding process. Two main commercial alloys from the Mg–Al–RE system, i.e., the AE42 and AE44 alloy (precisely, Mg-4Al-2.5RE-0.1Mn and Mg-4Al-3.9RE-0.3Mn, respectively), are the most common [47,48], although extensive research is being carried out on many experimental alloys with different ratios of aluminium to rare earth elements. Most of these studies concern the description of the primary microstructure (in as-cast conditions), including that obtained after the high-pressure casting process [35,48,49]. Nevertheless, it should also be noted that recent research also concerned the welding processes of various different magnesium alloys with rare-earth elements [50,51,52,53]. This work presents for the first time the results obtained after GTA welding of the new experimental alloys (from the Mg-Al-RE-type system with RE/Al ratios equal to 0.6 and 1) in order to illustrate both the possibility of applying the process to them and describing the obtained microstructure.

## 2. Materials and Methods

Two experimental alloys (Mg-5Al-3RE and Mg-5Al-5RE) with 5 wt% aluminium and 3 and 5 wt% rare earth elements were used in this study. The chemical composition of the investigated alloys is given in Table 1. Rare earth elements in the form of cerium-rich mischmetal (the composition according to the attestation was 54.8 wt% Ce, 23.8 wt% La, 16 wt% Nd, 5.4 wt% Pr, 0.16 wt% Fe, and 0.19 wt% Mg) were used. Additionally, a low volume fraction (i.e., 0.4 wt%) of manganese in magnesium with aluminium alloys was also used in order to reduce the iron content (as a harmful impurity) in the casts. The alloys were gravity-cast in steel moulds under the same conditions. The gas tungsten arc (GTA) welding process was conducted by means of a Falting 315 AC/DC instrument (OZAS, Opole, Poland) on a clean cast plate (free of oil and grease) of the dimensions 140 × 25 × 15 mm. The tungsten electrodes used in this study had a diameter of 2.4 mm (WT20 according to the DIN Standard). The shielding gas was helium with a flow rate of 20 L/min. The investigations were conducted utilizing the following welding parameters: 13 V voltage, 250 A current, and 12 mm/s welding speed.

**Figure 2:**
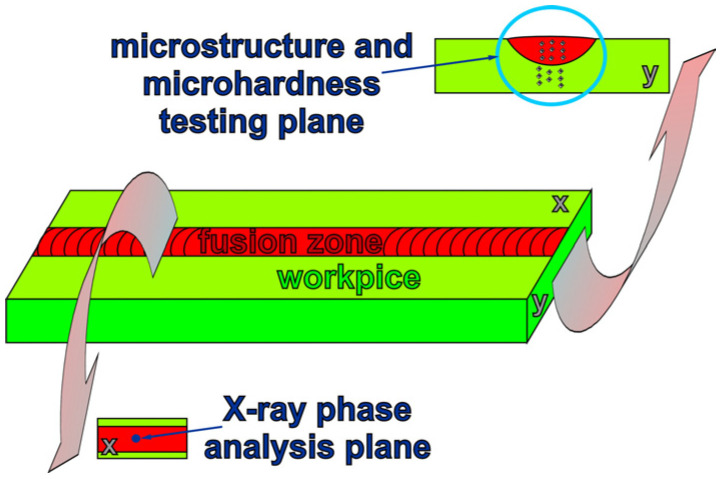
Scheme of researched areas.

A Brucker D8 Advance diffractometer (Bruker Corporation, Billerica, MA, USA) with CuKα X-ray radiation was employed in order to ascertain the phase composition of the investigated alloys after GTA welding. Reflexes from particular phases were identified according to ICDD PDF-4ţ cards [54]. For the GTA-welded materials, X-ray diffraction patterns were obtained from the fusion zone areas perpendicular to their depth, according to Figure 2.

Microstructure observations of the alloys in the initial stage and after GTA welding were carried out by means of a JOEL JSM-6610LV scanning electron microscope (SEM) (JOEL Ltd., Tokyo, Japan) with an energy-dispersive X-ray spectrometer (EDX) (Oxford Instruments, Abingdon, UK). The specimens for the microstructure investigations were prepared by standard metallographic procedures and, to reveal the microstructure, the specimens were etched in a 1% solution of HNO3 in C2H5OH for about 60 s.

The linear method of stereology was also used in order to determine the DAS (dendrite arm size) parameter, which can describe the microstructure changes caused by the GTA welding process. In this case, due to the kind of obtained microstructure, the DAS parameter corresponded to the average α-Mg phase size. Additionally, Vickers microhardness was determined by means of a Future-Tech FM-7 microhardness tester (Future-Tech Corp., Kawasaki, Japan) (load: 490.3 mN; time: 10 s). Average values were calculated based on 12 measurements for each material.

## 3. Results and Discussion

**Figure 3:**
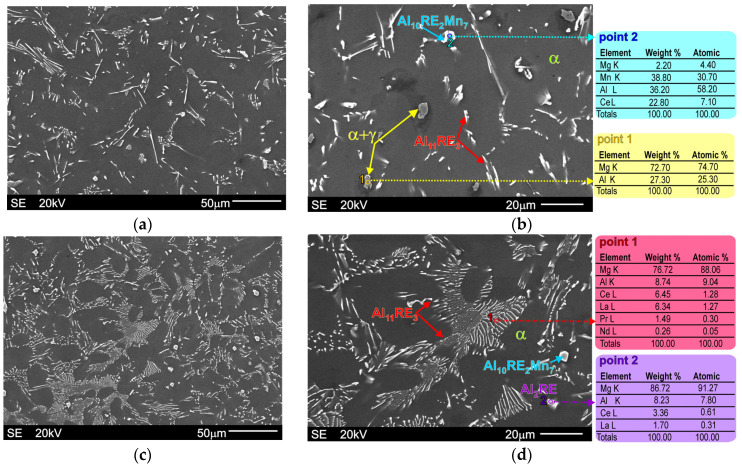
Microstructure of Mg-5Al-3RE alloy ((**a**,**b**): SEM micrographs taken at different magnifications) and Mg-5Al-3RE alloy ((**c**,**d**)**:** SEM micrographs taken at different magnifications) under as-cast conditions with EDX results obtained from designated points.

Figure 3 presents representative SEM micrographs of the microstructure of the investigated alloys in the initial stage (as-cast conditions). They consist of an α-Mg solid solution and an Al11RE3 intermetallic compound. In the microstructure of the Al-5Al-3RE alloy, a small volume fraction of the α+γ divorced eutectic is also visible, whereas this structural compound is practically below quantity sensitivity in the microstructure of the Al-5Al-5RE alloy. On the other hand, some amounts of the Al2RE intermetallic compound can be observed in the microstructure of the Al-5Al-5RE alloy, while this phase does not exist in the microstructure of the Al-5Al-3RE alloy. In the microstructure of both alloys, a ternary Al10RE2Mn7 intermetallic compound also formed owing to the presence of a low weight fraction of manganese in the chemical composition of the investigated materials.

The results presented in Figure 3 also illustrate the morphology of particular phases, which was identified by the EDX analyses. It should be noted, however, that the above-mentioned phases in the investigated alloys were also confirmed by XRD and TEM techniques because the high electron beam penetration during the EDX analyses of magnesium alloys prevents precise identification. The results of these examinations were presented in detail in previous works [34]. Nonetheless, thanks to the EDX results (presented in Figure 3) combined with the morphology analyses, a detailed description of individual structural constituents is possible. The Al11RE3 phase in the magnesium alloys has an acicular morphology, whereas the Al2RE and Al10RE2Mn7 intermetallic compounds are characterized by a blocky morphology [34,36]. As can also be easily seen by comparing Figure 3a,b and Figure 3c,d, with a decreasing Al/RE ratio, the volume fraction of the main Al11RE3 phase increases significantly. Additionally, the size of the DAS parameter also decreases, which is clearly visible during the comparison of Figure 3a with Figure 3c. The DAS parameters determined by quantitative metallography for the Al-5Al-3RE alloy was equal to 20.8 µm, while for the Al-5Al-5RE alloy, it was only 10.2 µm.

**Figure 4:**
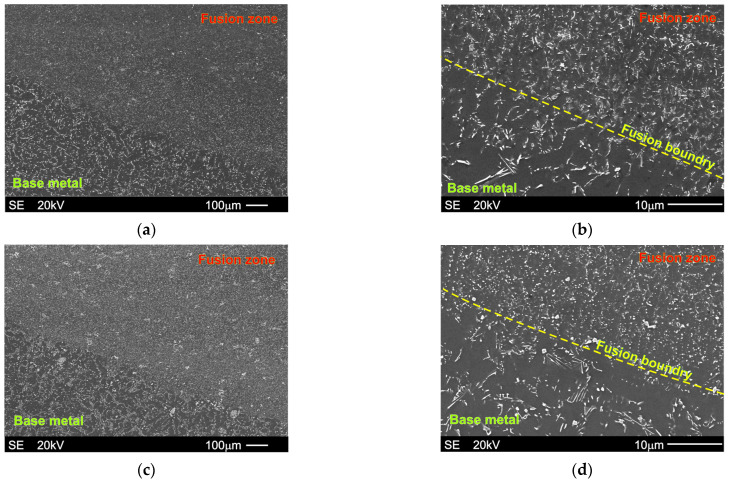
SEM micrographs presenting base metal, fusion boundary, and fusion zone after GTA welding of Mg-5Al-3RE alloy ((**a**,**b**): micrographs taken at different magnifications) and Mg-5Al-3RE alloy ((**c**,**d**): micrographs taken at different magnifications).

Figure 4a,b shows the SEM micrographs of the fusion boundary, fusion zone, and base material microstructure of the Mg-5Al-3RE alloy after GTA welding. Analogical images of the Mg-5Al-5RE alloy microstructure after GTA welding are presented in Figure 4c,d. In both cases, the fusion boundary is clearly visible.

**Figure 5:**
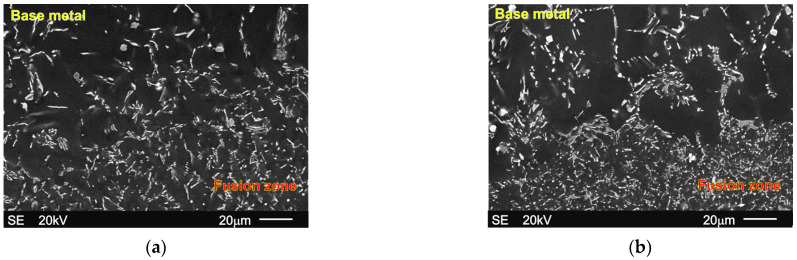
Microstructure of fusion boundary after GTA welding of Mg-5Al-3RE alloy (**a**) and Mg-5Al-5RE alloy (**b**), SEM.

Additionally, the fusion boundaries for both alloys are also presented at higher magnification in Figure 5a,b. It should also be noted that for the presented experimental alloys, distinct partially melted zones were not observed. In Mg–Al-type alloys, a wide partially melted zone (PMZ), located from the fusion boundary to the base metal, is observed very often [15,17,26,28,29], resulting from the low-melting point nature of the eutectic transformation, TE, of only 710 K (and also of the γ phase—about 723 K). For non-uniform solidified Mg-Al-type alloys, the difference between the liquidus (TL) and solidus temperatures (TE) is equal to about 463-433 K (depending on the Al weight fraction), which has an influence on the heat distribution below the fusion zone. Generally, in the partially melted zones, the α+γ eutectic is remelted and resolidified (varying in morphology), whereas the central areas of primary α-Mg dendrites do not change [26]. PMZ was also observed by Wagner et al. [13] in the Mg-6Zn alloy, which is also characterized by a very wide solidification range. In the presented Mg-5Al-3RE and Mg-5Al-5RE alloys, the γ phase is suppressed by the formation of mainly the Al11RE3 intermetallic phase. The calculated equilibrium liquidus temperature (according to ThermoCalc data [54]) is equal to 891 K for the Mg-5Al-3RE alloy and 888 K for the Mg-5Al-5RE alloy. The solidus temperature (according to the equilibrium condition) is 844 and 855 K for the Mg-5Al-3RE and Mg-5Al-5RE alloys, respectively. Therefore, the solidification range for the investigated alloys is considerably narrower (i.e., only 47–33 K) than for binary Mg-Al- or Mg–Zn-type alloys. Hence, the material was remelted only in the fusion zone.

**Figure 6:**
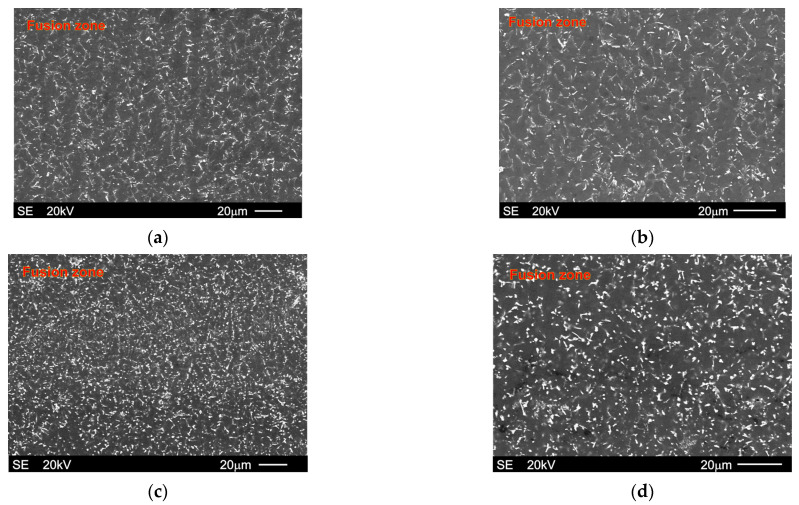
Microstructure of Mg-5Al-3RE alloy ((**a**,**b**): micrographs taken at different magnifications) and Mg-5Al-3RE alloy ((**c**,**d**): micrographs taken at different magnifications) after GTA welding process (areas form fusion zones), SEM.

**Figure 7:**
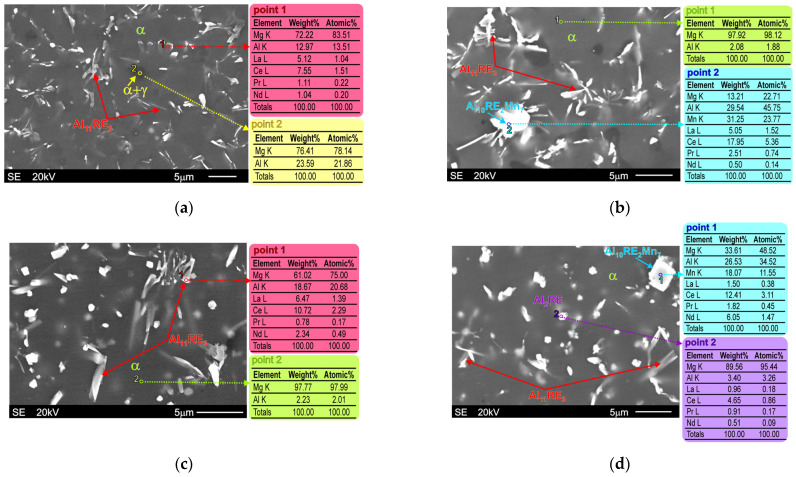
SEM micrographs of Mg-5Al-3RE alloy ((**a**,**b**): SEM micrographs taken from different areas of fusion zone) and Mg-5Al-3RE alloy ((**c**,**d**): SEM micrographs taken at different areas of fusion zone) after GTA welding process with EDX results obtained from designated points.

Figure 6 displays the microstructure in the fusion zones of the investigated materials after the GTA welding process. It is clearly visible that the microstructure of both examined alloys in the fusion zone has a dendritic morphology. A higher magnification of the SEM allowed a detailed observation of particular phases, as shown in Figure 7, which also presents the results of EDX analyses from designated points marked on the relevant microstructure micrographs.

**Figure 8:**
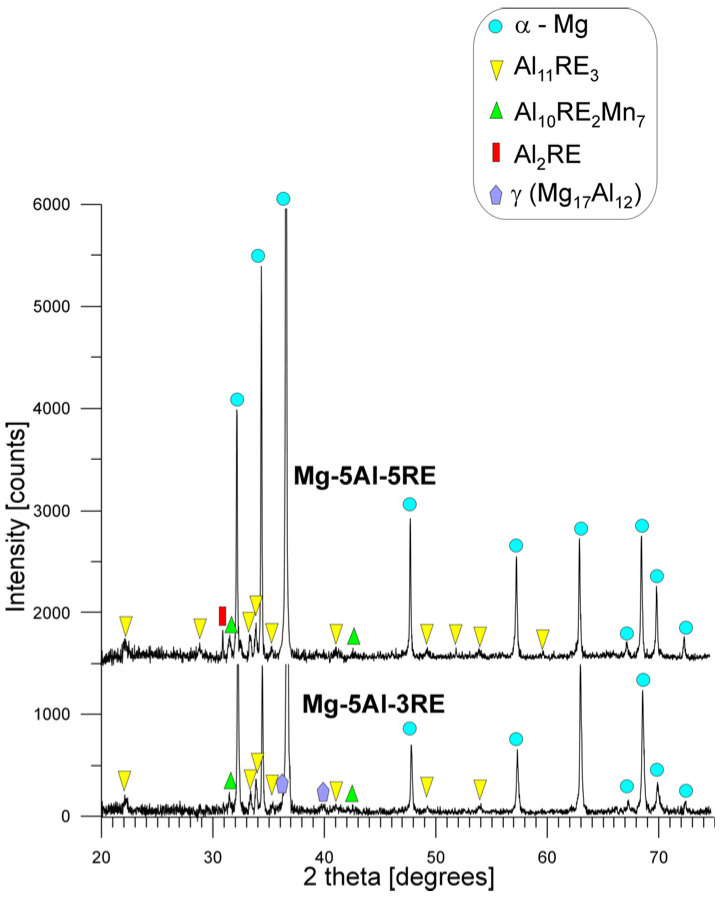
X-ray diffraction patterns of Mg-5Al-3RE and Mg-5Al-5RE alloys after GTA welding process (from fusion zones).

As a consequence of the high width and depth of the electron beam penetrating the magnesium alloys during the investigations, the obtained results from the EDX analyses are limited by some errors, such as the presence of magnesium in every examined point. In spite of this, based on the analyses of the presence of individual elements in the analysed structural constituents and their morphology, it is possible to identify the individual phases. Nevertheless, XRD investigations were carried out to confirm the phase composition of the fusions zones, the results of which are presented in Figure 8. The X-ray diffraction patterns obtained from the fusion zones did not reveal different phases to those observed in the base metals either, and confirmed the same structural constituents. During these analyses, the individual intermetallic compounds were identified based on the following phases: Al_11_Ce_3_ phase (Immm space group, a = 0.4389 nm, b = 1.0072 nm, and c = 1.3011 nm [54]), Al_2_Ce phase (Fd-3m space group, a = b=c = 0.8015 nm [54]), and Al_10_Ce_2_Mn_7_ phase (R-3m space group, a = b = 0.9040 nm, c = 1.3170 nm [54]).

**Figure 9:**
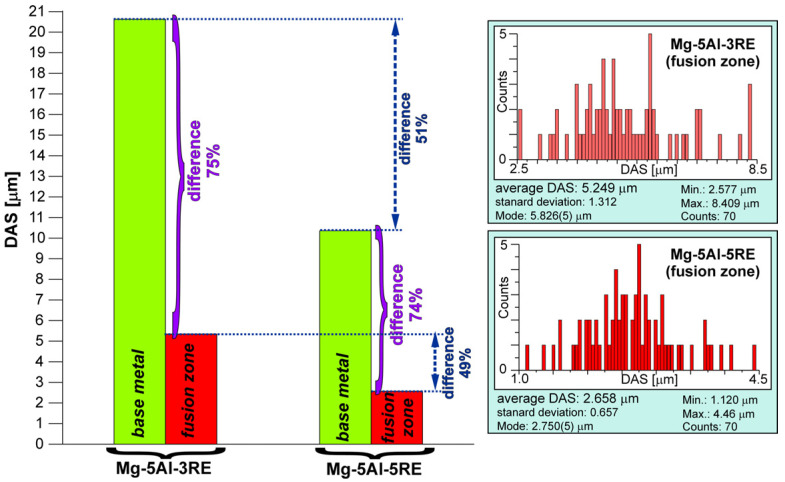
Comparison of DAS parameter values of investigated alloys in as-cast conditions and after GTA welding process with detailed results obtained from fusion zones of Mg-5Al-3RE and Mg-5Al-5RE alloy.

By comparing the microstructure of the base metal (Figure 3) and fusion zone shown in Figure 6, it is also clearly visible that the dendrite arm size (DAS) was substantially reduced after GTA welding. The growth of dendrites in the fusion zones was hampered by the rapid cooling during GTA welding. Additionally, the cooling speed was induced by the good thermal conductivity and low capacity of magnesium alloys [26]. Figure 9 presents the differences in the DAS parameter between the base metal and fusion zone for both investigated alloys. As stated above, the difference in the DAS parameter between both studied alloys in as-cast conditions was 51%. The same influence of rare earth elements on the DAS values is also visible in the alloys after GTA welding. The difference in the DAS parameter between the welded alloys was 49% and the Mg-5Al-5RE alloy also exhibited a lower DAS value than the Mg-5Al-3RE alloy after GTA welding. It is well known that the DAS parameter depends on the solidification conditions, especially the cooling rate. Because both alloys were cast and GTA-welded under the same conditions, the influence of rare earth elements on the DAS value is unequivocal. Nonetheless, the GTA welding process caused a decrease in DAS by 74-75% in comparison to the as-cast investigated alloys.

Additionally, it can be noted that the dendrite arm size in the fusion zone of both experimental alloys was smaller than those obtained for the commercial Mg-Al alloy (AZ91) welded using the same GTA parameters, which was presented in a previous study [26]. On the other hand, the size of the other structural constituents was also significant reduced. The Al_11_RE_3_ intermetallic phase was visibly significantly finer in the fusion zones than in the base metals for both investigated alloys. Nonetheless, it should also be noted that the microstructure of the researched alloys after the GTA welding process was more homogeneous than that obtained after the high-pressure die casting process (in which a biomodal grain size distribution occurs very often). Nevertheless, the Mg-5Al-5RE alloy showed a greater microstructure refinement than the Mg-5Al-3RE alloy after both the GTA welding process presented in this paper and the high-pressure die-casting process described in [55]. Analogical relationships were obtained for different parameters described in [56].

**Figure 10:**
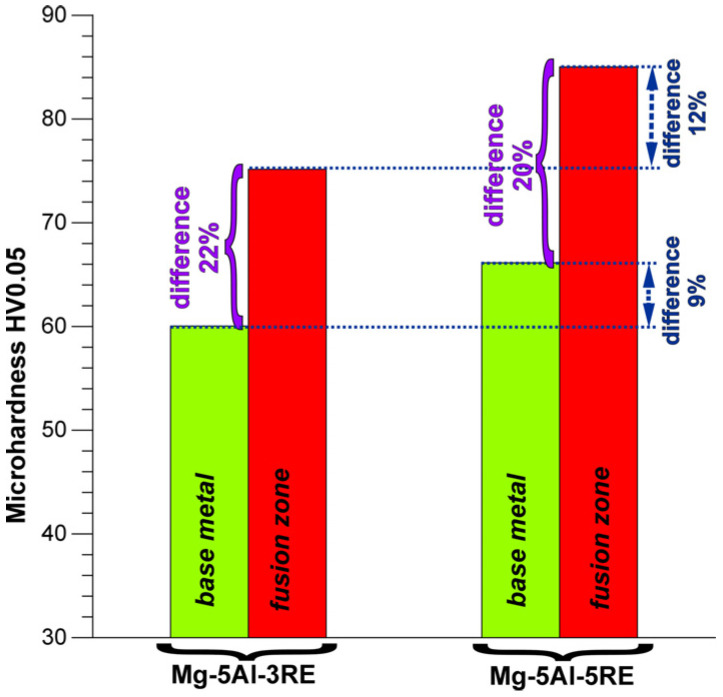
Comparison of microhardness values of investigated alloys in as-cast conditions and after GTA welding process.

Both the volume fraction of the Al_11_RE_3_ phase and the size of the microstructural constituents also affected in direct proportion the Vickers microhardness of the alloys. Figure 10 presents the differences between the HV0.05 values for the investigated alloys in as-cast conditions and after the GTA welding process. An increase in the mass fraction of rare earth elements in the Mg-5Al-3RE alloy in as-cast conditions caused a rise in this property by 12% in comparison to the Mg-5Al-3RE alloy. The same dependence between the microhardness values and rare earth elements in the alloys was observed in the fusion zone. The microhardness measured in the fusion zone of the AME505 alloy was 9% higher than in the fusion zone of the AME503 alloy. After the welding process, the Mg-5Al-3RE alloy had a microhardness equal to 75 HV0.05, whereas this property for the Mg-5Al-5RE alloy was equal to 85 HV0.05. The standard deviations of these values were 3.1 and 2.7 HV0.05, respectively. However, it should be noted that the GTA welding process caused growth in the microhardness by about 21% in comparison to the base metal for both investigated alloys. The obtained dependencies show that for the studied alloys, the degree of microstructure refinement (obtained after the GTA welding process) is also a significant factor influencing microhardness.

The presented results of analyses of the two experimental alloys from the Mg-Al-RE-system indicate that this type of magnesium alloy can be successfully surface-remelted by the gas tungsten arc welding process. Both alloys exhibited the same influence of the GTA welding process on the microstructure refinement and microhardness. Although the obtained growth in the microhardness of the Mg-5Al-3RE and Mg-5Al-5RE experimental alloys was not very high (about 21%), it should be taken into consideration that this result corresponds to those received for different magnesium alloys. Future studies should investigate in detail the influence of the GTA welding process on the corrosion resistance and tribological properties of this type of alloy.

## 4. Conclusions

The influence of the GTA welding process on the microstructure characterization of the Mg-5Al-3RE and Mg-5Al-5RE experimental alloys was investigated and the following results were revealed:

1. Two experimental Mg-Al-RE-type alloys were successfully GTA-welded and exhibited an intensive structure constituent size reduction. The DAS parameter in the melted zone for both alloys was about 75% lower than for the base metal.

2. The chemical composition of the examined alloys influenced the solidification range and prevented the formation of a partially melted zone observed in binary Mg–Al- or Mg–Zn-type alloys.

3. The GTA welding process caused an increase in microhardness by about 21% in comparison to the base metal for both investigated alloys.

## Figures and Tables

**Table 1:** Nominal chemical composition of investigated magnesium alloy.

Alloy	Chemical Composition wt%			
	Al	RE	Mn	Mg
Mg-5Al-3RE	5	3	0.4	balance
Mg-5Al-5RE	5	5	0.4	balance

## Data Availability

The original contributions presented in this study are included in the article. Further inquiries can be directed at the corresponding author.
